# Characterisation of Major Histocompatibility Complex Class I in the Australian Cane Toad, *Rhinella marina*

**DOI:** 10.1371/journal.pone.0102824

**Published:** 2014-08-05

**Authors:** Mette Lillie, Richard Shine, Katherine Belov

**Affiliations:** 1 Faculty of Veterinary Science, University of Sydney, Sydney, New South Wales, Australia; 2 School of Biological Sciences, University of Sydney, Sydney, New South Wales, Australia; Johns Hopkins University, United States of America

## Abstract

The Major Histocompatibility Complex (MHC) class I is a highly variable gene family that encodes cell-surface receptors vital for recognition of intracellular pathogens and initiation of immune responses. The MHC class I has yet to be characterised in bufonid toads (Order: Anura; Suborder: Neobatrachia; Family: Bufonidae), a large and diverse family of anurans. Here we describe the characterisation of a classical MHC class I gene in the Australian cane toad, *Rhinella marina*. From 25 individuals sampled from the Australian population, we found only 3 alleles at this classical class I locus. We also found large number of class I alpha 1 alleles, implying an expansion of class I loci in this species. The low classical class I genetic diversity is likely the result of repeated bottleneck events, which arose as a result of the cane toad's complex history of introductions as a biocontrol agent and its subsequent invasion across Australia.

## Introduction

The cane toad (*Rhinella marina*, formerly *Bufo marinus*) is a large, toxic anuran native to Mexico, Central and tropical South America, and the island of Trinidad [1]. The family Bufonidae is large and widespread, including 485 species of 35 genera, with representative species naturally occurring on all continents, except Australasia and Antarctica [2]. During the 1930s, the cane toad was a popular biological control agent for invertebrate agricultural pests and was introduced to numerous countries including Australia [1]. One hundred and one individuals were introduced in 1935 and bred for distribution to agricultural districts of north-eastern Australia [1]. They quickly became well established and began invading into northern and eastern Australia [1]. The cane toad population in Australia currently covers more than 1 million square kilometres and continues to spread [3].

The Major Histocompatibility Complex (MHC) is a large gene complex present in all jawed vertebrates with an integral role in the immune system. The antigen-presenting molecules encoded by the MHC class I and class II genes are cell-surface glycoproteins that bind intracellular and extracellular peptides, respectively [4]. The class I molecule consists of an α chain with 3 extracellular domains and a β2 microglobulin. Peptides derived from the degradation of intracellular pathogens are bound into a peptide-binding groove formed by the α1 and α2 domains [5]. Anchor residues in the peptide binding groove determine the specific peptides that can be bound by a particular MHC class I molecule. These peptide-MHC complexes are presented to cytotoxic T-lymphocytes and an appropriate immune response is launched on recognition of foreign bound peptides [4].

The MHC genes evolve under a birth and death model [6]. Multiple gene copies are generated through duplication events (“birth”). These two copies are then able to diverge and assume distinct peptide binding specificities or entirely new functions. Some of the duplicates may become pseudogenized by nonsense or frameshift mutations and “die”. Through the course of this gene evolution, the numbers of MHC loci vary greatly within different species.

The class I genes are categorised as either classical or non-classical based on their functional differences. Classical MHC class I molecules are expressed on the surface of all nucleated cells, are highly polymorphic and are involved in presentation of self and non-self peptides to cytotoxic T cells [4]. The non-classical class I loci have conventionally been described as having variable expression in tissues, typically have low polymorphism, and have a variety of functions within the immune system [7]; although studies of classical and non-classical class I loci in human and mouse are challenging these definitions [8].

In anurans, it has long been held that there is clear distinction between the classical and non-classical loci. In the model anurans, *Xenopus laevis* and *Silurana tropicalis*, a single classical class I locus has been characterised in each species, along with a large expansion of non-classical class I loci [9], [10], [11], [12]. These non-classical genes have low polymorphism and differ in their invariant amino acids in the peptide-binding groove, which determines their peptide anchoring [13]. A single classical class I locus was also identified in *Rana pipiens* [14] and *R. temporaria* [15]. But recently, multiple putatively classical class I loci have been identified in several anuran species, with at least 3 loci identified in species representing the family Hylidae, 2 loci in a species representing Centrolenidae and 3 loci in other species within the family Ranidae, within which *R. pipiens* and *R. temporaria* lie [16].

The MHC class I gene has yet been characterised in a bufonid species. Here we present the characterisation of the cane toad MHC class I gene from a spleen cDNA library and its comparison to other species. Primers were then designed to characterise allelic variation at the α1 domain and α2 domain in the genomic DNA of 25 individuals.

## Materials and Methods

### Purification of nucleic acids

*Xenopus laevis* genomic DNA was purified from denuded oocytes using DNeasy Blood and Tissue Kit (QIAGEN) with proteinase K digestion at 56°C for 1.5 hr. Oocyte manipulations followed a protocol approved under the Australian Code of Practice for the Care and Use of Animals for Scientific Purposes. Cane toad toe samples were collected at four locations (Cairns, 16°55′S, 145°46′E, colonised 1936; Normanton, 17°40′S, 141°04′E, colonised 1966; Borroloola, 16°04′S, 136°18′E, colonised 1988; Timber Creek, 15°38′S, 130°28′E, colonised 2006) across the cane toad distribution. Genomic DNA was purified from these toe sample using DNeasy Blood and Tissue Kit (QIAGEN) with proteinase K digestion at 56°C for at least 3 hrs. All protocols were carried out under permits issued by the Northern Territory Parks and Wildlife Commission and with approval of the University of Sydney Animal Care and Ethics Committee (L04/5-2008/2/4790 and L04/11-2006/3/4478). Euthanasia was carried out with pentobarbital with all efforts made to minimise animal suffering.

Total RNA was purified from cane toad tissues using RNeasy Plus mini Kit (QIAGEN) following the manufacturer's protocol. These were DNase treated prior to cDNA synthesis using SuperScript III First-Strand Synthesis System (Invitrogen).

### Construction and screening of cDNA library

A cDNA phage expression library was constructed from a cane toad spleen RNA using the PCR-based SMART cDNA Library Construction Kit (Clontech). Size fractionated (>100 bp) cDNA were ligated into the λTriplEx2 arms (Clontech) and packaging with a GigaPack III Gold extract kit (Stratagene). Primary titres for the unamplified libraries were determined to be approximately 5×10^6^ pfu/ml. The libraries were then amplified by transducing *Escherichia coli* XL-1 Blue with phage suspensions according to the manufacturers protocol (Clontech).

PCR primers (XelaMHCIa F and XelaMHCIa R; also see table 1) were designed from *X. laevis* MHC Class I sequences available on Genbank to amplify the conserved alpha 3 (exon 4) domain. The 181 bp fragment was amplified from *X. laevis* DNA using PCR in a volume of 20 µl containing 1×PCR buffer (Invitrogen), 2.5 mM MgCl, 0.2 mM dNTPs, 0.8 mM each primer, and 0.125 U *Taq* polymerase (Invitrogen).

**Table 1:** PCR primers.

Species	Primer names	Primer sequence (5′-3′)	T_A_	Product length
*Xenopus laevis*	XelaMHCIa F	AATCAGATGACGCCACAGA	56	181
*Xenopus laevis*	XelaMHCIa R	CCTCATTGGGTGTAATCTCAG	56	
*Rhinella marina*	Class I_Alpha1domF	ACAGTCACTCTCTGCGTTATT	60	251
*Rhinella marina*	Class I_Alpha1domR	GTTGAAGCGGCTCATC	60	
				241
*Rhinella marina*	RhmaA1altF3	TCTGCGTTATTATGTAACTGG	55	
*Rhinella marina*	Class I_Alpha2domF	GGATGTACGGCTGTGAGCTG	60	243
*Rhinella marina*	Class I_Alpha2domR	GATCTTCTCTCCCGTGCTCCA	60	

PCR products were confirmed by electrophoresis on an agarose gel consisting of 2% agarose, 1×TBE (89 mM Tris base, 89 mM boric acid, 2 mM EDTA, pH 8) and 1×SYBRsafe (Invitrogen) run in 1×TBE electrophoresis buffer. PCR products were excised from the gel and purified using a QIAEX II Gel Purification Kit (QIAGEN) according to the manufacturer's protocol. These PCR products were cloned using the pGEM-T Easy Vector System (Promega) and purified using QIAprep Spin Miniprep Kit (QIAGEN) prior to sequencing on a ABI 3730 sequencer at the Australian Genome Research Facility (AGRF, Westmead, Australia). Plasmids confirmed to contain *X. laevis* MHC Class I alpha 3 were used as the DNA template for amplification of the probe for screening the cDNA library.

Plaques were lifted from culture plates using Amersham Hybond N+ hybridisation membranes (GE Healthcare). Membranes were then soaked in denaturation solution (1.5 M NaCl, 0.5 M NaOH) for 7 min, neutralising solution (1.5 M NaCl, 0.5 M Tris, 1 mM EDTA, pH 7.2) for 3 min, washed in 2xSSC (0.3 M NaCl, 0.03 M Trisodium citrate) for 5 min then fixed at 80°C for 2 hr. The MHC Class I alpha 3 probe was radioactively labelled using Random Primed DNA Labelling Kit (Roche Applied Science) and [α-^32^P]dCTP. Unincorporated dNTPs were removed using Illustra ProbeQuant G-50 Micro Columns (GE Healthcare). After overnight hybridization at 55°C, the membranes were washed and subjected to autoradiography overnight or longer at −80°C. Positive plaques were isolated through primary and secondary screenings.

Plaque phages were converted to plasmid by in vivo excision in *E. coli* BM25.8 following the SMART protocol. 5 µl was used to plate on LB agar with 100 µg/ml ampicillin. Isolated colonies were picked and used to inoculate LB broth for overnight incubation followed by plasmid clean up with QIAprep Spin Miniprep Kit (QIAGEN). Purified plasmid DNA was sequenced by AGRF (Westmead, Australia) with the SMART 3′ or 5′ sequencing primers (Clontech).

### MHC class I allelic diversity

The cane toad MHC class I full length transcript was aligned to the *Xenopus laevis* MHC class I gene (accession no.: M58019; [17]) to predict exons. Two sets of PCR primers were designed to amplify the α1 and the α2 domain using Oligo 6 (Molecular Biology Insights, Inc.) to investigate allelic diversity in 25 cane toad individuals. These primer binding sites were located in highly conserved regions of the MHC class I regions as identified by sequence alignments with *Xenopus* spp and *Rana* spp. Amplification was carried out in a total volume of 20 µl containing 1× HotStar HiFi PCR Buffer (QIAGEN), 1 µM each primer, 1 U Hotstar HiFidelity DNA Polymerase (QIAGEN) in a thermal cycler with an initial denaturation and activation of the polymerase at 95°C for 5 min, 35 cycles of 94°C for 30 s, 60°C for 1 min, 72°C for 45 s, and a final extension at 72°C for 10 min.

Amplification was confirmed on a 2% agarose/TBE gel. PCR products were excised from the gel and purified using the QIAEX II gel purification kit (QIAGEN). Purified products were ligated into a pGEM-T Easy vector (Promega) prior to transformation of competent JM109 *Escherichia coli* cells (Promega). Transformants were plated onto LB/agar plates supplemented with 100 µg/ml ampicillin, 0.5 mM IPTG and 80 µg/ml X-Gal to allow for blue/white screening and incubated overnight. Positive clones were picked and cultured in LB broth with 50 µg/ml ampicillin overnight prior to plasmid purification using a QIAprep Spin Miniprep Kit (QIAGEN). Purified plasmids were sequenced by AGRF (Westmead, Australia) with the T7 sequencing primer. Twelve clones were collected per PCR, and allele sequences were accepted if they were observed in more than one individual or isolated from two independent PCR reactions.

### Sequence analysis

Sequences were edited in BioEdit 7 [18] and confirmed to contain MHC sequences using tblastx (available online at http://blast.ncbi.nlm.nih.gov/Blast.cgi; [19]). Codons were aligned by ClustalW as implemented in MEGA5 [20]. MEGA was also used to construct maximum likelihood phylogenies from the nucleotide sequences, using General Time Reversible model of nucleotide substitution as recommended by Modelgenerator [21] and rooted on their midpoints.

## Results and Discussion

### Isolation and characterisation of MHC class I alpha chain

Approximately 4.8×10^9^ pfus of the cane toad spleen cDNA library were screened with the *X. laevis* MHC class I α3 domain probe. After primary screening, 18 positive plaques were picked and cultured for secondary screening. 9 positive plaques were picked, converted to plasmid and sequenced. Partial sequences obtained from five plasmids yielded identical sequences with significant similarity to amphibian MHC class I using tblastx. Two positive plasmids were chosen for complete sequencing of both forward and reverse strands; both plasmids contained identical sequences.

**Figure 1:**
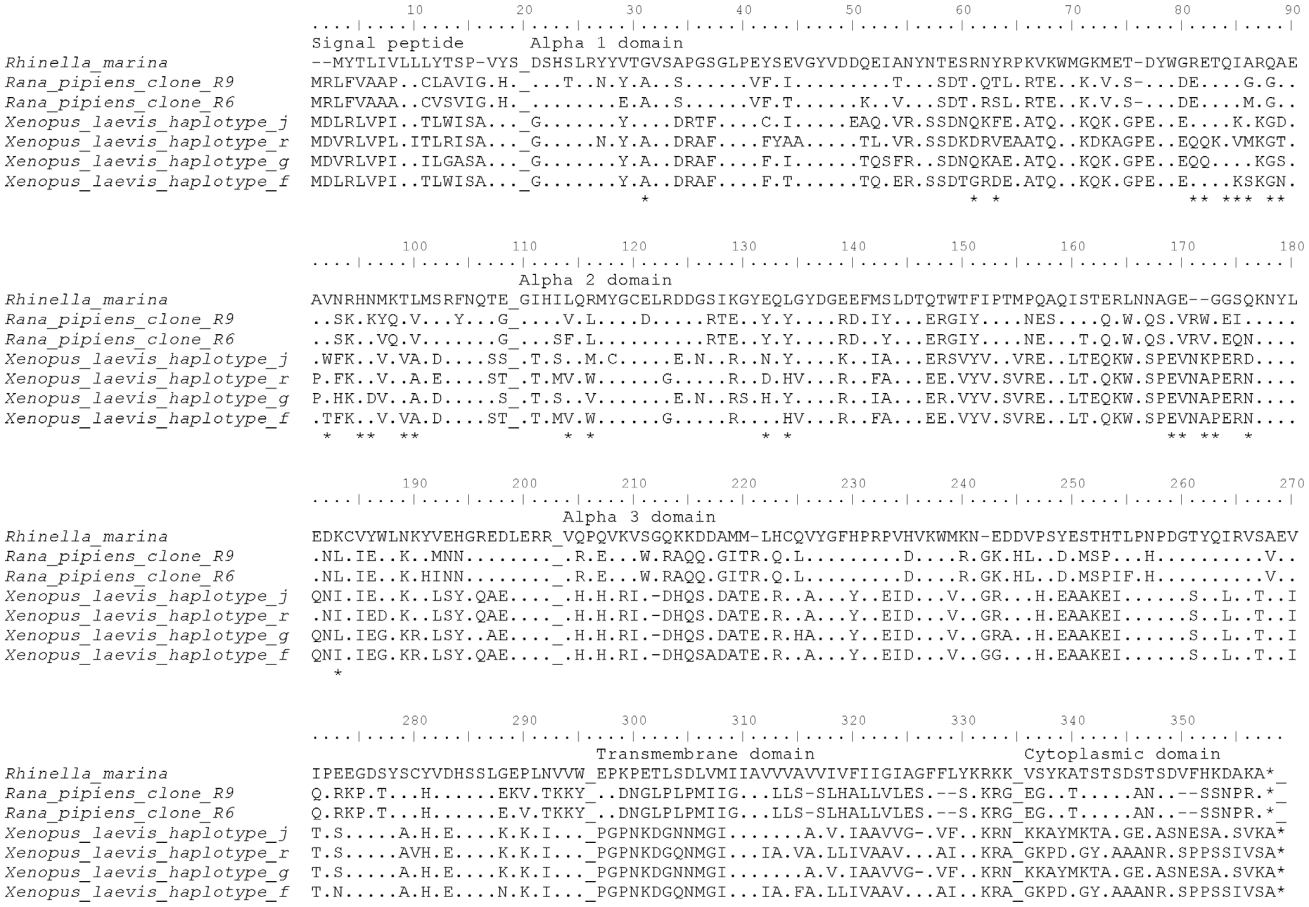
Amino acid alignment of anuran MHC class I. ClustalW alignment of MHC class I from Rhinella marina, Rana pipiens (MHC class I clone R6 Genbank accession no.: AF185587 and R9: AF185588) and Xenopus laevis (MHC class I haplotype g: AF188579; haplotype f: AF188580; haplotype r: AF188582 and haplotype j: AF188586); * indicate putative peptide binding residues as predicted from Flajnik et al. 1999.

The cane toad MHC transcript (Genbank accession: KC295548) was aligned to MHC class I genes of other anuran species (*X. laevis* MHC class I haplotype g: AF188579; haplotype f: AF188580; haplotype r: AF188582 and haplotype j: AF188586; *R. pipiens* MHC class I clone R6: AF185587 and R9: AF185588). This alignment was used to predict the domains of the expressed MHC class I gene and the putative peptide binding residues (Figure 1).

**Figure 2:**
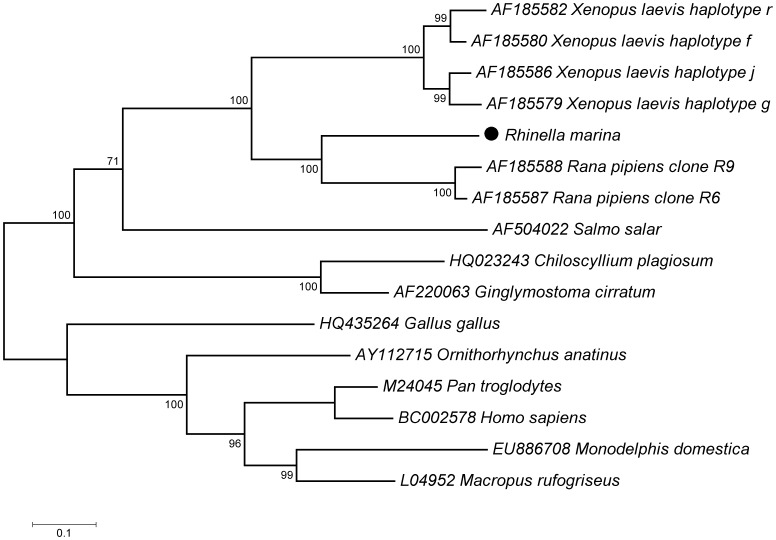
Phylogenetic tree comparing *Rhinella marina* MHC class I to other vertebrates. Maximum likelihood phylogenetic tree using General Time Reversible model of nucleotide substitution with invariant sites (rooted on midpoint) of MHC class I in cane toad, *Rhinella marina*, African clawed frog, *Xenopus laevis*, northern leopard frog, *Rana pipiens*, Atlantic salmon, *Salmo salar*, whitespotted bamboo shark, *Chiloscyllium plagiosum*, nurse shark, *Ginglymostoma cirratum*, chicken, *Gallus gallus*, platypus, *Ornithorhynchus anatinus*, gray short-tailed opossum, *Monodelphis domestica*, red-necked wallaby, *Macropus rufogriseus*, common chimpanzee, *Pan troglodytes*, and human, *Homo sapiens*. Sequence titles include Genbank accessions.

The sequenced cane toad MHC class I transcript was 1035 bp in length, containing an open reading frame of 344 amino acids, spanning the signal peptide, alpha 1, 2 and 3 domains, and the transmembrane and cytoplasmic domains (Figure 1). It had on average 64% nucleotide similarity and 53% amino acid similarity with the MHC class I of *R. pipiens*, and only 57% nucleotide and 44% amino acid similarity with *X. laevis*. Phylogenetic analysis revealed the cane toad MHC class I clustered together with class I sequences from *R. pipiens* as a sister clade to MHC class I sequences from *X. laevis* (Figure 2).

### MHC class I α1 and α2 domain polymorphism

Two sets of primers were designed from the full-length transcript to assess the allelic variation in the alpha 1 domain and the alpha 2 domain. Specific residues encoded by these domains are involved with peptide binding and have been shown to be highly polymorphic in many species because they are under the influence of positive selection [22], [23]. PCR primers were designed to maximise the number of codons predicted to have peptide binding function from an alignment with *X. laevis* [14] and primer binding sites were localised to regions that appeared highly conserved across anuran lineages.

**Figure 3:**
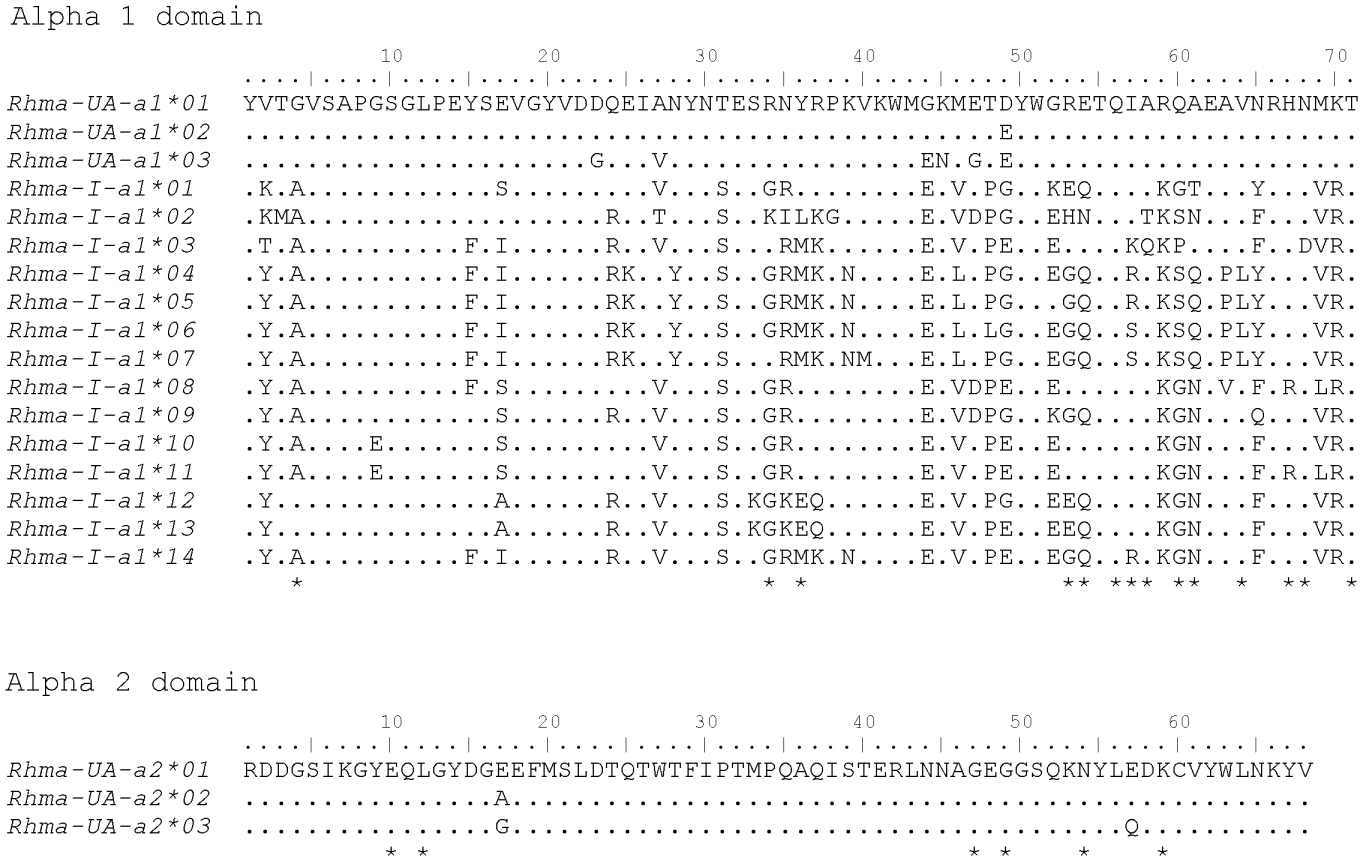
Amino acid alignment of MHC class I alpha 1 domain and alpha 2 domain variants. Putative peptide binding sites are indicated with * under each alignment.

**Figure 4:**
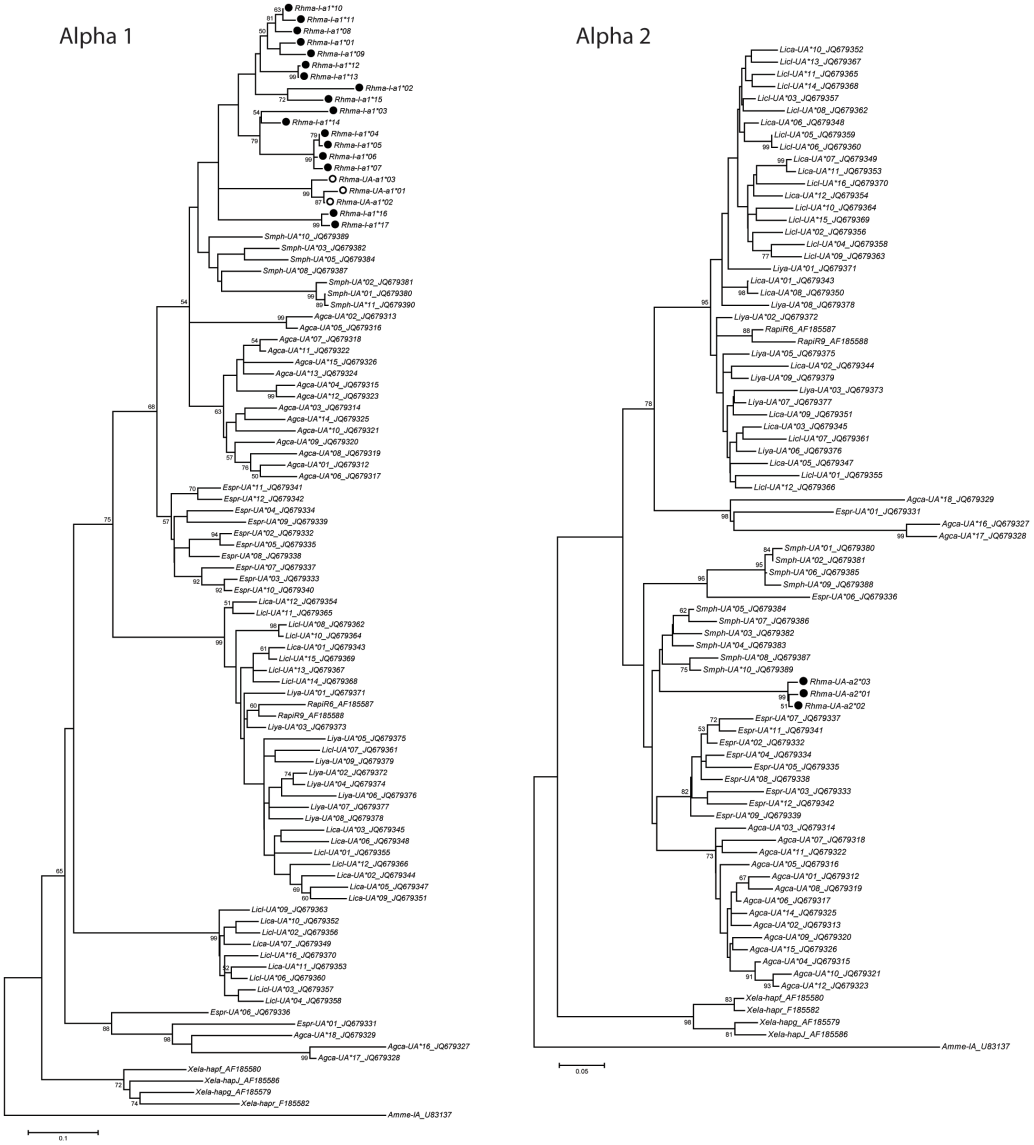
Phylogenetic trees for MHC class I alpha 1 domain and alpha 2 domain. Maximum likelihood phylogenetic trees using General Time Reversible model of nucleotide substitution with invariant sites (rooted on midpoint) comparing *Rhinella marina* (Rhma) to other anuran species (*Xenopus laevis*, Xela; *Silurana tropicalis*, Sitr; *Rana temporaria*, Rate; *Agalychnis callidryas*, Agca; *Espadarana prosoblepon*, Espr; *Smilisca phaeota*, Smph; *Lithobates catesbeianus*, Lica; *L. clamitans*, Licl; *L. yavapaiensis*, Liya) and axolotl (*Ambystoma mexicanium*, Amme). Sequence titles include Genbank accessions.

The alpha 1 PCR primers (Class I_Alpha1domF and Class I_Alpha1domR; Table 1) amplified a 214 bp fragment that included 14 putative peptide-binding sites. Up to 8 different variants were sequenced in one single individual, indicating that these primers amplify at least 4 loci. From the 25 individuals, 17 alpha 1 variants were characterised (Figure 3; Genbank accessions: KC295549-KC295565). These variants share between 73% to 99.5% nucleotide sequence similarity and between 54.9% and 98.5% amino acid sequence similarity. Allele variant Rhma-UA-a1*01 was identical to the full length transcript and had between 94.4–98.1% sequence similarity to Rhma-UA-a1*02 and *03. These variants form a strongly supported phylogenetic clade (Figure 4) and a maximum of two alleles were found in each individual, supporting our conclusion that these alleles are from the *Rhma-UA* locus characterised from the cDNA library. The other alpha 1 allele variants may represent other alleles from other classical loci, alleles from non-classical loci, or pseudogenes. As classification and locus assignment of these alleles was beyond the scope of this study, we named them *Rhma-I-a1* (“I” for MHC class I, “a1” for alpha 1 domain, and numbered in order of characterisation).

The alpha 2 PCR primers (Class I_Alpha2domF and Class I_Alpha2domR; Table 1) amplified a 202 bp fragment, containing 6 putative peptide-binding sites. A maximum of 2 variants were isolated per individual, indicating the primers were specific for one class I locus. A total of 3 alleles were sequenced from 25 individuals (Figure 3; Genbank accession: KC295569-KC295571), which had between 98% and 99% nucleotide sequence similarity, and between 97% and 98.5% amino acid sequence similarity. Allele variant Rhma-UA-a2*01 was identical to the full length transcript and other allele variants were numbered in order of characterisation.

Phylogenetic trees constructed for the MHC class I alpha 1 and alpha 2 domains (Figure 4) show the cane toad allele variants forming a monophyletic clade. In both cases, they form a sister clade to class I sequences isolated from the Hylid frog, *Smilisca phaeota*. Both species represent the Hyloidea superfamily that diversified during the Paleogene, and are estimated to have last shared a common ancestor 66 MYA [24].

### Classification of *Rhma-UA-a1* allele variants

Our results indicate a large expansion of class I loci in the cane toad. Our aims were to characterise a classical class I locus for future population genetic surveys, as these typically are the most polymorphic class I loci. As such, we focused on the full length transcript characterised from the cDNA library and the alleles Rhma-UA-a1*01, *02, and *03 that belong to this locus.

As a preliminary investigation into whether Rhma-UA-a1*01-03 were indeed classical class I alleles, we compared the alpha 1 variants expressed in 7 different tissues, including liver, lung, kidney, heart, ventral skin, dorsal skin and spleen from one individual to the alpha 1 variants amplified from its genomic DNA. We expected that the classical class I alpha 1 variants would be highly expressed in all tissues, and as a result we would observed these variants at the highest frequency. Twenty clones were sequenced per tissue and 40 clones were sequenced from the genomic DNA. Results are summarised in table 2.

**Table 2:** Numbers of allele variants sequenced from cDNA from various tissues and the genomic DNA of one cane toad individual from Normanton, Australia.

	Rhma UA-a1*01	Rhma-I-a1 *02	Rhma-I-a1 *03	Rhma-I-a1 *04	Rhma-I-a1 *07	Rhma-I-a1 *08	Rhma-I-a1 *09	Rhma-I-a1 *12	Rhma-I-a1 *15	Rhma-I-a1 *16	Rhma-I-a1 *17
gDNA	14	4	1	3	2	5	1	2		3	3
Spleen	7	1	1		1	3	4				
Ventral Skin	14					1	2	1	1	1	
Lung	12	1	3				4				
Liver	8	2	2			2	2	3			
Dorsal Skin	17	1		1				1			
Kidney	7		3			2	4	1	1		
Heart	18	1						1			

Forty clones were sequenced from genomic DNA and 20 clones were seqeunced from each tissue type. Recombinant clones were identified and removed from genomic DNA (2), kidney (2), liver (1) and spleen (3) records.

Eleven alpha 1 variants in total were sequenced across all tissues and genomic DNA. An additional 3 alpha 1 variants (Rhma-I-a1*15–17; Genbank accession: KC295566-KC295568), previously uncharacterised, were isolated in this sequencing. The Rhma-UA-a1*01 variant was the most highly represented sequence isolated across all tissues. The remaining alpha 1 variants had variable expression patterns throughout the tissues, indicating that these variants may be non-classical. Although the Rhma-I-a1*02, *09, *12 variants showed a wide tissue distribution, their sequence frequency were lower than those of Rhma-UA-a1*01, supporting this notion. Further work to classify the non-classical allele variants and assign loci will provide valuable insight into the organisation of the cane toad MHC class I. With 10 putative non-classical alpha 1 variants sequenced from this one individual, it appears as though there are at least 5 class I loci in the cane toad in addition to the classical class I *Rhma-UA* locus. Characterisation of full-length transcripts will help to ascertain whether all these putative non-classical allele variants are functional genes, or pseudogenes, and allow locus assignment. Expression analysis will also help to classify these alleles as either classical or non-classical, based on their differential expression patterns.

It is worth noting that our primers could preferentially amplify the classical class I locus over other variants. The primers were designed from the full-length MHC class I transcript, which shared over 90% similarity to any *Rhma-UA-a1* allele. If there were mismatches in the primer-binding site, amplification of the *Rhma-I-a1* variants could occur at a lower frequency and lead to an underrepresentation of the putative non-classical allele variants in our cloning and sequencing.

We have subsequently sequenced a cane toad spleen transcriptome (Illumina HiSeq paired-end library, ABySS assembly, custom blast database, blastn and tblastx queries with our full length transcript and our allele variants). Contigs with significant similarity to the cane toad alpha 1 allele variants were extracted and aligned. This alignment revealed that the sequence at the ClassI_Alpha1domR priming site was 100% conserved between all UA allele variants. The sequence at the ClassI_Alpha1domF priming site was also highly conserved with a few exceptions. One C-T mismatch was identified on allele variants Rhma-I-a1*03, *12, and *08, however as these appeared in our cloning and sequencing, we can assume that our primers do bind and amplify these allele variants. We also observed a previously uncharacterised allele variant in the transcriptome with two C-T mismatches at the priming site, which may imply lower primer binding and subsequently low or no amplification of this allele variant. No other class I allele variants were observed in the transcriptome using these methods. Comparison of the alpha 2 domain primer-binding sites reveals that the primer sequences were highly specific to the *Rhma-UA* locus, which serves to explain why these primers did not amplify other MHC class I loci and the absence of non-classical alpha 2 domain allele variants in our cloning and sequencing results.

A new forward primer specific for the *Rhma-UA* alpha 1 locus was designed (RhmaAIaltF3; Table 1) that allows the amplification of a 204 bp fragment (together with the original reverse primer, Class I_Alpha1domR).

### Genetic variation of MHC class I α1 and α2 domains

The MHC class I is typically highly polymorphic in natural populations, but we only found 3 alpha 1 variants and 3 alpha 2 variants at *Rhma-UA* in 25 Australian cane toads. This is exceptionally low MHC class I diversity when compared to similar studies in other species (Table 3). Furthermore, these sequences were highly similar with between 94.4% and 98.1% nucleotide similarity at the alpha 1 domain and between 98% and 99.5% at the alpha 2 domain.

**Table 3:** Comparison of allelic diversity at the MHC class I (N is number of individuals studies and NA is number of alleles).

*Species and Citation*	*Class I domain/s studied*	*# loci*	*N*	*NA*
**Cane toad (** ***Rhinella marina*** **) this study**	**Alpha 1**	**1**	**25**	**3**
	**Alpha 2**	**1**	**25**	**3**
Common frog (*Rana temporaria*) [15]	Alpha 1 and 2	1	66	129
Sockeye salmon (*Oncorhynchus nerka*) [32]	Alpha 2	1	>100	34
Atlantic salmon (*Salmo salar*) [33]	Alpha 1,2, 3	1	82	11
Banded houndshark (*Triakis scyllium*) [34]	Alpha 1,2, 3 (Trsc-UAA)	1	22	29
	Alpha 1,2, 3 (Trsc-UBA)	1	22	6
Blue tit (*Cyanistes caeruleus*) [35]	Alpha 2	4	20	17
House Sparrow (*Passer domesticus*) [36]	Alpha 2	≥3	8	20
House Sparrow (*Passer domesticus*) [37]	Alpha 2	≥3	10	27

This severe depletion of MHC class I variation is the likely result of the history of serial translocations events, which gave rise to the modern Australian cane toad population. The repeated founder events drastically reduced genetic diversity across the entire cane toad genome. Only a single mitochondrial haplotype is found in the Australian population, which was identical to that found in their source population of Hawaii [25].

Relaxed balancing selection on the MHC may have further depleted variation in the MHC of the Australian cane toad. Prior to their introduction, bufonid toads were absent from Australia, and presumably so were the pathogens that have coevolved with them. The most effective pathogens of the Australian cane toad may be those that were co-introduced, but surveys of Australian and Hawaiian cane toad have demonstrated that they lack many of the parasites common in the natural populations in South America [26].

One South American parasite that was introduced to Australia with the cane toad is a lungworm, *Rhabdias pseudosphaerocephala*, which causes morbidity and mortality in a wide range of toad life-history stages [27], [28]. This parasite lags behind the cane toad invasion front [29], and thus the selection pressure it mediates across the population would be variable. Indeed it appears as though selection for dispersal traits has led to reduced immune function on the invasion front [30], [31]. Further research will investigate whether this translates into reduced selection pressure for MHC polymorphism leading to depletion of MHC diversity over the course of the cane toad invasion.

In conclusion, we characterised a single classical MHC class I gene in the cane toad and we infer an expansion of non-classical MHC class I loci from sequencing of alpha 1 domain allele variants. We found low MHC variability in Australian cane toads; the likely result of repeated bottleneck events during its history of introductions. This low MHC variability may indicate an immunological weakness of this invasive species, which may give hope to the development of a bio-control strategy.
